# Oral Health Practices of Dental Professionals Towards Self-Oral Care: A Cross-Sectional Study

**DOI:** 10.7759/cureus.94835

**Published:** 2025-10-17

**Authors:** Sumit Kumar, Gaurav Mishra, Vinay Kumar Gupta, Pramod Yadav, Nishita Kankane

**Affiliations:** 1 Public Health Dentistry, Faculty of Dental Sciences, King George's Medical University, Lucknow, IND; 2 Community Dentistry, Dr. Ziauddin Ahmad Dental College, Aligarh Muslim University, Aligarh, IND

**Keywords:** cross-sectional study, dental professionals, oral hygiene, self oral care, toothbrush force

## Abstract

Background: Dentists are considered positive role models and play a vital part in imparting effective oral health knowledge to patients and communities. Hence, a study was conducted to assess the attitude and practices of dental professionals towards self-oral care and their knowledge of toothbrushing force, which is widely not discussed.

Methods: A cross-sectional study was conducted among 309 dental professionals, including undergraduates, interns, postgraduates, senior residents, faculty, and private practitioners in Lucknow, India, from January 2024 to June 2024 using a semi-structured self-administered questionnaire.

Results: The mean age of the participants was 28 years, with more females (195; 63.11%) than males (114; 36.89%). A total of 240 (77.67%) participants used soft-bristle toothbrushes, and 233 (75.40%) used fluoridated toothpaste. The most common brushing technique was the modified bass technique (234; 75.73%). More than half of the participants always considered and applied the correct force during brushing from their point of view, while the rest did not consider it. About three-fourths were unaware of assessing and implementing brushing force, while the remaining 81 participants provided various responses, including subjective self-evaluation, flaring of bristles due to excessive pressure, injury to gum/enamel/teeth, and using sensing devices. Female participants were better than their male counterparts in terms of following a few oral hygiene practices regularly, in terms of using fluoridated toothpaste, and in the context of skipping toothbrushing in the morning and tobacco practices.

Conclusion: The study provided valuable insights for dental professionals regarding their attitudes and practices towards oral hygiene. The unique part of the study was that it focused on getting details on assessing and implementing the toothbrush force.

## Introduction

Good oral health is characterized by the absence of disease, and the ability to perform normal functions of the mouth is considered an integral component of overall health and well-being. In contrast, poor oral health can have a detrimental effect on the individual’s health [1,2]. As per the World Health Organization's oral health profile of India in 2022, the prevalence of untreated caries in deciduous teeth (children aged one to nine years) and permanent teeth (above five years of age) was 43.3% and 28.8%, respectively. At the same time, 21.8% of people above the age of 15 years had severe periodontal disease [3]. Multiple risk factors contribute to oral diseases, including increased use of sugar, oral hygiene habits, tobacco use (smoking/smokeless), stress, diet, and many more. These are also common risk factors for other chronic diseases such as diabetes, obesity, cardiovascular diseases, and cancer. Instead of one disease-specific approach, implementing an integrated common risk factor approach by Sheiham et al. will help in controlling these risk factors and will have a great impact on the majority of diseases, thus promoting health in general [4].

Oral diseases can limit day-to-day functions, decrease the quality of life, and reduce overall productivity due to pain, loss of work hours, daily performance, and income. The expenditure on prevention and treatment also has a long-term impact on one’s socioeconomic status, ultimately affecting the nation's economy [5]. The total dental healthcare expenditure in 2019 was 64 million US$ in India. In the same year, total productivity loss due to five oral diseases was 7249 million US$ [3].

Dental health professionals are considered positive role models and play a vital part in imparting effective and evidence-based oral health knowledge to patients and communities, thereby influencing their oral health behaviour. Therefore, if these professionals practice the ideal way of self-oral healthcare, it will have a lasting impact on patients and communities, ultimately leading to better oral health in society [6-8].

These practices include correct brushing technique, adequate duration, sufficient frequency, the right toothpaste, interdental aids, and ideal bristle hardness. If not followed correctly, it may contribute to gingival recession, non-carious cervical lesions, and other oral health conditions [9]. Therefore, oral healthcare professionals are key players in creating a positive environment for good oral health in society through promoting these practices [10].

It is often observed that dental professionals have sufficient knowledge about maintaining good oral hygiene, but many of them fail to follow these practices adequately due to a lack of proper attitude or insufficient time. Additionally, how much force should be applied while cleaning teeth with a toothbrush is not discussed widely.

Hence, this study was conducted to assess the attitude and practice of dental professionals towards self-oral care as well as their consideration of toothbrushing force.

## Materials and methods

This was a descriptive cross-sectional study conducted among dental care providers, including undergraduates, interns, postgraduates, senior residents, faculty, and private practitioners (referred to as participants), in Lucknow, India. The study was conducted from January 2024 to June 2024.

A convenience sampling method was used, whereby all eligible and consenting dental professionals available during the study period were invited to participate. Although the study used convenience sampling, a priori estimation was done using the single proportion formula, $n = \frac{Z^2 \cdot p \cdot (1-p)}{d^2}$, with Z = 1.96, 𝑝 = 0.50 and 𝑑 = 0.06. Allowing 10% for potential non-response. The final sample used in the study was 309. Participants above the age of 18 years who gave consent were included in the study. Those with active orthodontic treatment and not willing to participate were excluded from the study.

Ethical approval from the Institutional Ethical Committee was obtained before the commencement of the study (Ref. Code: 127th ECM IIA/P20; Letter No. 3263/Ethics/2024). The study ensured compliance with ethical standards by obtaining written informed consent from all the participants. Additionally, to ensure anonymity, the survey did not collect any identifiable information from the participants.

A semi-structured self-administered questionnaire was prepared in the English language to collect information from the study participants. It was a mix of single-choice, multiple-choice, and open-ended questions assessing the attitude and practice of participants for self-oral care. A new subjective aspect is explored in this research, i.e., toothbrushing force. Since there is no practical method to assess the toothbrushing force objectively, the question about the measurement of toothbrushing pressure was not kept in the scope of this research rather it was tried to assess whether the subjects consider the brushing force or not from subject's point of view by asking question, “Do you always consider the application of appropriate force while brushing your teeth?” The preliminary questionnaire was tested through a pilot study among 10% of the desired sample size. The preliminary questionnaire consisted of a total of 26 items. Most questions were categorical multiple-choice items (nominal variables such as yes/no, type of aid, etc.). Some were ordinal frequency-based items (e.g., always/sometimes/never; once/twice/thrice). One question was open-ended (how participants assess brushing force). Content and face validity were assessed to ensure that the questionnaire covered the study objectives and was clear and adequately understood by participants. The responses were also checked for ambiguity, comprehension, and the appropriateness of the language, along with the words used. Based upon the pilot study, six items were rejected due to redundancy, lack of clarity, and irrelevance to the study objectives and minor corrections were made in three questions, resulting in a final 20 items for the questionnaire. The questionnaire was also checked for internal consistency. Cronbach’s alpha was used to find the reliability coefficient, which was found to be 0.766. Subjects were contacted during their relatively free time, which was previously scheduled. Data were obtained by face-to-face interviews using the validated questionnaire. Survey responses were exported to Excel and pre-coded by a researcher. After cleaning and final checks, the data were analysed using STATA 15.1 (StataCorp LLC, College Station, TX). P < 0.05 was considered statistically significant. Descriptive analysis was performed, and Fisher's exact and chi-square tests were used to assess the association between different variables. Associations between categorical variables were tested using Pearson’s chi-square test when all expected cell counts were ≥5; otherwise, Fisher’s exact test was used.

## Results

A total of 309 dental professionals participated in the study. Most participants were 21 to 30 years old, and 55.66% were postgraduate students. The mean age of the participants was 28.02 ± 5.10 years. There were more females (195; 63.11%) than males (114; 36.89%). Table 1 represents the sociodemographic characteristics of the participants.

**Table 1:** Sociodemographic characteristics of the participants (n = 309). Data are presented as numbers (n) and percentages (%).

Characteristics	Number (n)	Percentage (%)
Age category		
21-30 years	262	84.79
31-40 years	32	10.36
41-50 years	13	4.21
51-60 years	2	0.65
Gender		
Male	114	36.89
Female	195	63.11
Occupation		
Undergraduate student	27	8.74
Intern	52	16.83
Postgraduate student	172	55.66
Faculty	18	5.83
Private practitioner	10	3.24
Senior resident	30	9.71

A total of 240 (77.67%) participants used soft-bristle toothbrushes, and fluoridated toothpaste was used by 233 (75.40%) participants. The most common brushing technique among participants was the modified bass technique (234; 75.73%), followed by mixed/scrub.

Most participants (230; 74.43%) brush their teeth twice daily, preferably in the morning before breakfast and before retiring to bed. Only 28 (9.06%) participants skipped brushing their teeth in the morning hours, citing time constraints. A total of 216 (70%) of participants changed their toothbrush after three to four months. Mouthwash was occasionally used by participants (186; 60.19%). This shows that the majority of dental professionals follow proper oral hygiene practices regularly.

Less than half (149; 48.22%) of the participants rinse their mouth with water after every snack, indicating a somewhat ignorant attitude towards one of the good oral practices.

Out of all, 150 (48.54%) of participants did not use any additional aid to clean the interdental area, while 104 (33.66%) used only dental floss. Of those participants who were using dental floss, only 43 (41.35%) used it correctly.

The majority of the participants had no dental problems, and only 41 (13.27%) participants had some form of oral health problems. Of those having dental problems, 18 (43.9%) participants were suffering from dental problems for the last six months, followed by 10 (24.39%) for more than one year.

Around three-fourths of the participants who had dental problems had undergone treatment. The majority of the participants (37; 90%) who did not undergo any treatment for their dental problems cited their busy schedule as a deterrent.

Further, most of the participants (279; 90.29%) mentioned undergoing oral prophylaxis as well as staying away from tobacco consumption (253; 81.88%). This aligns with the expectation that dental professionals, being non-tobacco users, set a positive example in promoting oral health, which increases their credibility in educating the public about the harmful effects of tobacco use.

Almost all the participants felt that their own oral health may affect their patients’ oral health. When patients see their dental providers having good oral hygiene, they usually listen to them attentively and try to follow their advice.

**Figure 1:**
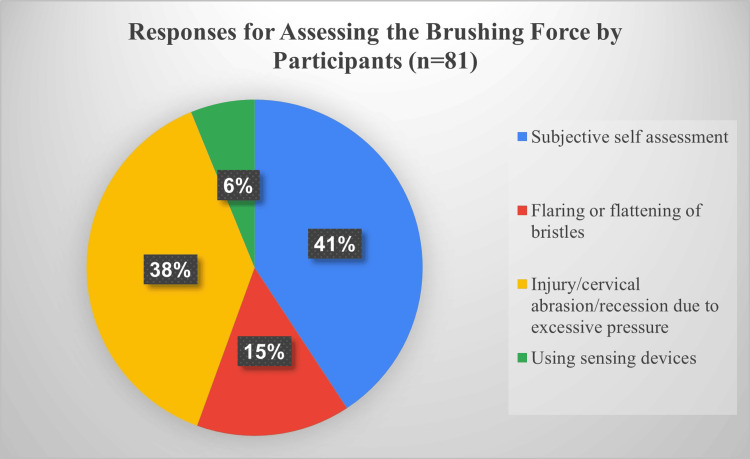
Distribution of responses for assessing brushing force used by the participants (n = 81). Data are presented as a pie chart.

More than half of the participants (175; 56.63%) quoted that they consider the application of appropriate force during brushing, while 134 (43.37%) did not consider it. This disparity raised concern about whether participants were aware of correct force application. To explore it further, an open-ended question was asked to know how they practically assess and implement the toothbrush force in daily brushing. A total of 228 (73.79%) participants did not respond about brushing force, while the remaining 81 (26.21%) participants provided various responses. Thematic analysis was done for the responses. The themes for responses we got were “subjective self-evaluation”, “flaring of bristles due to excessive pressure”, “injury to gum/enamel/teeth”, and “using sensing devices” (Figure 1).

Table 2 represents Fisher's exact and chi-square analyses showing differences in oral hygiene practices among males and females. A significant difference was found between males and females in toothpaste choice (p = 0.021). There were also differences in skipping morning brushing, with more males skipping compared to females (p = 0.020). Males reported a higher percentage of oral problems compared to females (p = 0.041). The analysis also highlights the significant difference in tobacco use, with males using tobacco more frequently.

**Table 2:** Association of oral hygiene practices among males and females (n = 309). This table depicts the association of oral hygiene practices among males and females using the chi-square and Fisher's exact tests. Statistical significance was defined as p < 0.05.

Oral hygiene practices	Gender		Test value	P-value (level of significance)
	Male, n (%)	Female, n (%)		
Which toothpaste do you use?			Fisher's exact = 0.021	0.021
Fluoridated	82 (35.19)	151 (64.81)		
Non-fluoridated	17 (34.00)	33 (66.00)		
Herbal	6 (40.00)	9 (60.00)		
I don’t consider	9 (81.82)	2 (18.18)		
Do you sometimes skip brushing your teeth in the morning hours?			Pearson's chi-square= 5.4228	0.020
Yes	16 (57.14)	12 (42.86)		
No	98 (34.88)	183 (65.12)		
Do you have any oral/dental problems at present?			Pearson's chi-square = 4.1673	0.041
Yes	21 (51.22)	20 (48.78)		
No	93 (34.70)	175 (65.30)		
Do you use tobacco in any form (smoking/smokeless)?			Fisher's exact = 0.000	0.000
Yes (habitual)	6 (100.00)	0 (0)		
Yes (occasional)	12 (80.00)	3 (20.00)		
I used to, but I have quit	8 (22.86)	27 (77.14)		
I never used	88 (34.78)	165 (65.22)		

A significantly large proportion of postgraduates had undergone oral prophylaxis (57.71%) compared to undergraduates (p = 0.018).

## Discussion

There were 309 participants enrolled in the study, with a mean age of 28 years, with more females than males. Most participants used soft-bristle toothbrushes and fluoridated toothpaste. The predominant brushing technique among participants was the modified bass technique, which is a widely used technique and considered effective for plaque removal and gum protection. Around three-fourths of participants brush their teeth twice daily, preferably in the morning before breakfast and before retiring to bed. The majority of them change their toothbrushes every three to four months.

Less than half of the participants used to rinse their mouth with water after every snack regularly. Around half of the participants did not use any interdental aid. Almost all the participants had undergone oral prophylaxis, and a high rate of non-tobacco users was reported among participants.

In this study, the mean age of participants was 28.02 ± 5.10 years, which was younger than the mean age reported by Ghasemi et al. (2007) [11], where the mean age was 37.4 ± 7.7 years. There were more females (195; 63.11%) than males (114; 36.89%) in the present study, which was consistent with the findings of the study by Riad et al. (2022) [6], where 66.5% were females and 33.5% were males. Contradict to the present study, there were 56.3% males and 44.2% females in the study by Kumar et al. (2011) [8].

There were relatively more participants (230; 74.43%) who brushed twice daily in the present study compared to the studies by Reddy et al. (2012) [12] and Kumar et al. (2011) [8], where only 34.91% and 45.8% of participants, respectively, brushed their teeth twice daily. A study by Yao et al. (2019) [2] reported a higher prevalence of 93.2%, which was comparatively higher than the present study.

Additionally, 233 (75.40%) participants regularly used fluoridated toothpaste for brushing in the present study, while, in contrast, only 44.58% used fluoridated toothpaste in the study by Reddy et al. (2012) [12] and relatively less (32.6%) in the other study [8]. This shows dental professionals were better aware of following regular oral hygiene practices in the present study.

Flossing of the teeth is essential for complete cleanliness of interdental areas. Only 5.6%, 10.6%, and 14.2% used dental floss in cleaning the interdental area in a few studies [2,8,12]. In contrast, 104 (33.66%) used only dental floss to clean their interdental areas in the present study. Still, there is a need to educate professionals regarding the benefits of flossing.

The most common method of toothbrushing was the modified bass technique (234; 75.73%) in the present study, while only 20.5% participants used the above technique in the study by Yao et al. (2019) [2].

Almost all participants in the study by Kumar et al. (2017) [1] preferred soft toothbrushes over hard bristles. In the present study, the percentage is slightly less (240; 77.67%).

Only 23.33% of participants used mouthwash in addition to toothbrushing in one of the studies [1]. While in the present study, a comparatively large number of participants used mouthwash occasionally or regularly. The majority of participants were non-tobacco users in the present study, which was in concordance with the study by Reddy et al. (2012) [12].

The present study showed that female participants were better than their male counterparts in terms of following a few oral hygiene practices regularly. This may be attributed to their positive self-care attitudes, driven by internal psychological motivations such as enhancing appearance and self-esteem. This was in accordance with a few studies, including Kumar et al. (2017) [1] and Halboub et al. (2016) [13], and contrary to the study by Ahamed et al. (2015) [7].

One interesting finding, which was not observed in other studies, was that more than half of the participants quoted that they considered applying the appropriate force while brushing their teeth, while the rest did not consider it. In contrast, when it was discussed in detail with all the participants regarding how to practically assess and implement the toothbrush force, about three-fourths did not give any response, while the remaining 81 participants provided various responses. Thematic analysis was done for the responses. The themes for responses we got were “subjective self-evaluation”, “flaring of bristles due to excessive pressure”, “injury to gum/enamel/teeth”, and “using sensing devices”. The response “using sensing devices” is appropriate for the question, but the fact is that there is no sensing device available for manual toothbrushes which can be used by every person. This aspect of toothbrushing technique has never been considered to be the focus, in spite of the fact that an excessive amount of force exerted on teeth while brushing may lead to irreversible damage to hard and soft tissue.

Good oral healthcare practices are of crucial importance in everyone’s life, and dentists are no exception to it. In the current study, although the major proportion of participants followed good oral healthcare practices towards self-oral care but the participants being dentists, this proportion must be much higher. Although the data are not statistically significant for many variables but there is a significant reflection that not only the majority, rather each and every dentist must follow all possible ways to prevent the occurrence of any oral health problem. Most of the participants in the current study recognized the importance of the fact that their own oral health can influence their patients. This gives them an additional responsibility in the interest of society, and also to follow the ideal ways of oral care. In this regard, these facts must be quoted while addressing the attitudes of oral health professionals with respect to their own oral healthcare.

The unique part of the present study was that it focused on getting details on subjective assessment and consideration of appropriate toothbrushing force. The limited understanding of toothbrush force among dental health professionals indicates the need for further research in this area, and structured objective training and development of practical ways and sensing devices to guide patients about correct force application.

The study has certain limitations regarding the generalizability, with respect to a limited convenience sample, a single site, and no comparison group. Since the subjects are dental professionals, there could have been social desirability bias. The insights gathered from the study may be used to plan further research on some specific elements, like toothbrushing technique, association of oral health practices with overall health, and toothbrushing force.

## Conclusions

This study highlights the critical role of dental professionals in maintaining and promoting oral health standards within their communities. While a significant majority of participants demonstrated good oral hygiene practices, including the use of soft-bristle toothbrushes and fluoridated toothpaste, there remain notable gaps in their understanding and application of effective brushing force. This lack of awareness may inadvertently contribute to oral health issues, emphasizing the need for further education and training in this area.

Given these insights, it is essential to foster a culture of self-care among dental professionals, ensuring they model exemplary oral hygiene behaviours. Future research should focus on refining educational approaches regarding toothbrush force application, the development of cost-effective sensing devices compatible with manual toothbrushes, and integrating these practices into dental training programs. By empowering dental professionals as ambassadors of oral health, we can enhance community awareness and contribute to improved health outcomes on a larger scale.

## Appendices

Questionnaire used in the study

Section A - Demographic Details (Five Questions)

• Name (removed at the time of analysis)

• Age (categorized for analysis)

• Gender (Male/Female)

• Mobile number (removed at the time of analysis)

• Current status in the dental field

Section B - Oral Health Practices and Behaviours (21 Questions)

1. What kind of toothbrush do you use?

a. Soft

b. Medium

c. Hard

d. I don’t consider

2. Which toothpaste do you use?

a. Fluoridated

b. Non-fluoridated

c. Herbal

d. I don’t consider

3. Which technique do you most commonly use?

a. Bass

b. Modified bass

c. Charter’s

d. Mixed/scrub/other…………………..

4. How many times do you actually brush your teeth regularly?

a. Once

b. Twice

c. Thrice

d. After eating anything

5. What is the timing of tooth brushing in relation to food intake? (More than one response can be chosen)

a. Morning before breakfast

b. Morning after breakfast

c. Evening, right after dinner

d. Before retiring to bed

6. Do you sometimes skip brushing your teeth in the morning hours?

a. Yes

b. No

7. If yes, what may be the reason?

a. Because of a busy schedule

b. I forget sometimes

c. Any other (specify)………..

8. After how much time do you change your toothbrush?

a. After three to four months

b. More than six months

c. When visible flaring of bristles occurs

d. There is no such criteria for me

9. Do you use mouthwash?

a. Yes, regularly

b. Occasionally

c. No

10. Do you rinse your mouth with at least water, practically always after eating any snacks, etc.?

a. Yes, sometimes

b. Yes, always

c. No

11. What do you prefer to use for cleaning the interdental area daily?

a. I don’t need any additional aid to clean the interdental area daily

b. Tooth-pick

c. Dental floss

d. Interdental brush

e. Other (please specify)……………………..

12. If you use floss, what is the way you use it to clean the interdental area?

a. Up & down

b. Back & forth

c. Both

d. Haphazard

13. Do you have any oral/dental problems at present?

a. Yes

b. No

14. If yes, since when have you had the problem? (Answer only if your answer is yes in question 13)

a. Within one month

b. Within six months

c. Within one year

d. More than one year

15. If yes, have you taken the treatment? (Answer only if your answer is yes in question 13)

a. Yes

b. No

16. If no, why? (Answer only if your answer is no in question 15)

a. Afraid of dental procedures being done on myself

b. Because of a busy schedule

c. Because I know it will not increase further as I keep a watch on it

d. Other (please specify)……………………………………………………………….

17. Have you undergone oral prophylaxis for your teeth?

a. Yes

b. Never

18. Do you use tobacco in any form (smoking/smokeless)?

a. Yes (habitual)

b. Yes (occasional)

c. I used to, but I have quit

d. I never used

19. Do you feel that your good/poor oral health can impact your patients?

a. Yes

b. No

20. Do you always consider and practically apply the measurement of correct force application while brushing your teeth?

a. Yes

b. No

21. Practically, in daily life, how can the toothbrush force application be assessed and implemented?

Kindly write in your own words.

__________________________________________________________________

__________________________________________________________________

__________________________________________________________________
